# Novel compound heterozygous pathogenic variants in the *SLC3A1* gene in a Chinese family with cystinuria

**DOI:** 10.1186/s12920-023-01767-6

**Published:** 2023-12-19

**Authors:** Danhua Liu, Yongli Zhao, Xia Xue, Xinyue Hou, Hongen Xu, Xinghua Zhao, Yongan Tian, Wenxue Tang, Jiancheng Guo, Changbao Xu

**Affiliations:** 1 The Research and Application Center of Precision Medicine, The Second Affiliated Hospital, Zhengzhou University, Zhengzhou, 450000 China; 2 Department of Urology, the Second Affiliated Hospital of Zhengzhou University, NO. 2 Jingba Road, Zhengzhou, 450014 China; 3 Henan Key Laboratory of Helicobacter Pylori & Microbiota and Gastrointestinal Cancer, Marshall Medical Research Center, The Fifth Affiliated Hospital of Zhengzhou University, Zhengzhou, 450002 China; 4 Precision Medicine Center, Academy of Medical Sciences, Zhengzhou University, Zhengzhou, 450000 China; 5 Henan Institute of Medical and Pharmaceutical Sciences, BGI College, Zhengzhou University, Zhengzhou, 450052 China

**Keywords:** *SLC3A1*, Cystinuria, Novel variants, NGS, The Chinese population

## Abstract

**Background:**

Cystinuria is an autosomal recessive disorder characterized by a cystine transport deficiency in the renal tubules due to mutations in two genes: *SLC3A1* and *SLC7A9*. Cystinuria can be classified into three forms based on the genotype: type A, due to mutations in the *SLC3A1* gene; type B, due to mutations in the *SLC7A9* gene; and type AB, due to mutations in both genes.

**Methods:**

We report a 12-year-old boy from central China with cystine stones. He was from a non-consanguineous family that had no known history of genetic disease. A physical examination showed normal development and neurological behaviors. Whole-exome and Sanger sequencing were used to identify and verify the suspected pathogenic variants.

**Results:**

The compound heterozygous variants c.898_905del (p.Arg301AlafsTer6) is located in exon5 and c.1898_1899insAT (p.Asp634LeufsTer46) is located in exon10 of *SLC3A1* (NM_000341.4) were deemed responsible for type A cystinuria family. The variant c.898_905del was reported in a Japanese patient in 2000, and the variant c.1898_1899insAT is novel.

**Conclusion:**

A novel pathogenic heterozygous variant pair of the *SLC3A1* gene was identified in a Chinese boy with type A cystinuria, enriching the mutational spectrum of the *SLC3A1* gene. We attempted to find a pattern for the association between the genotype of *SLC3A1* variants and the manifestations of cystinuria in patients with different onset ages. Our findings have important implications for genetic counseling and the early clinical diagnosis of cystinuria.

**Supplementary Information:**

The online version contains supplementary material available at 10.1186/s12920-023-01767-6.

## Introduction

Cystinuria (OMIM 220100) is a congenital amino acid metabolic disorder that can lead to defective luminal transport of dibasic amino acids (cystine, ornithine, lysine, and arginine), and the defective reabsorption of cystine can lead to dramatically increased cysteine stone formation in the kidneys and other organs [8, 23, 27]. Cystine is a sulfur-containing homodimeric amino acid, which is composed of two cysteine molecules connected by disulfide bonds. Under physiological conditions, cystine is filtered freely by the glomerulus and reabsorbed by the proximal renal tubules, and its transport mechanism is not completely clear [5]. The transport proteins rBAT (encoded by *SLC3A1*) and b0, + AT (encoded by *SLC7A9*) participate in high-affinity transport of cystine and dibasic amino acids in kidney and intestine by forming a heterodimeric transport network connected by a covalent disulfide bond [3,  4, 10, 19, 21, 31]. Defects in transport protein cause impaired reabsorption of cysteine, which results in hyperexcretion of cystine in the urine [26], due to the low solubility of cystine in normal urine pH, patients tend to form cystine stones.

Cystinuria can be caused by mutations in the *SLC3A1* and/or *SLC7A9* gene [8, 14]. The *SLC3A1* gene is located on chromosome 2p16.3–21, and the *SLC7A9* gene is located on chromosome 19q13.11. In 2002, the International Cystinuria Consortium classified cystinuria based on mutations in different genes into type A (*SLC3A1* mutations), type B (*SLC7A9* mutations) and type AB (both the *SLC3A1* and *SLC7A9* mutations) [ 8, 13, 18, 27, 28]. A classification based on urinary amino acid excretion identified type 1 (normal cysteine excretion), type non-1 (moderate and high cysteine excretion), mixed, and unidentified cystinuria [14]. However, the correlation between the genotype and phenotype of cystinuria remains unclear. Cystinuria caused by *SLC3A1* mutations is usually an autosomal recessive disease and *SLC3A1* heterozygotes have no apparent phenotype, but cystinuria caused by *SLC7A9* mutations is usually autosome dominant inheritance with 86% of *SLC7A9* heterozygotes having abnormal urinary dibasic amino acid levels and some of them will develop into Cystine stones [28]. There is no difference between patients with the type A (biallelic *SLC3A1* mutations) and type B (biallelic *SLC7A9* mutations) in a variety of clinical parameters. However, carriers with a single heterozygous mutation also have different disease severity levels and cannot be distinguished from patients with biallelic *SLC3A1/SLC7A9* mutations [7, 12, 23]. The complex genetic pattern and incidence of clinical forms of cystinuria differ across populations. The complex genetic pattern and incidence of clinical forms of cystinuria differ across populations. The frequency of cystinuria is approximately as 0.000143 in neonates, 0.0004 among Libyan Jews, 0.0000667 in Americans, and 0.000001 in Swedes [30]; in Chinese nephrolithiasis patients, the frequency of cystine stones is 0.00005 [27, 32]. However, the exact frequency of cystinuria carriers in different populations remains unclear because there are limited records of the prevalence of this disease in different populations [8].

In 1994, pathogenic variants of the *SLC3A1* gene were identified as leading to cystinuria [3]. Currently, the Human Gene Mutation Database (HGMD® Professional 2022, http://www.hgmd.cf.ac.uk/ac/index.php, accessed December 4, 2022) contains nearly 260 pathogenic variants of *SLC3A1* reported from different populations. Almost 95% of biallelic *SLC3A1* mutations lead to cystine stone formation at some age, and the recurrence rate of stones and related metabolic diseases is as high as 60% [13]. Therefore, it is challenging to predict the phenotypes of individual cystinuria patients from different countries due to population-based variability [23, 25, 29, 30].

This study identified compound heterozygous variants of the *SLC3A1* gene (c.898_905del (p.Arg301AlafsTer6) and c.1898_1899insAT (p.Asp634LeufsTer46)) in a 12-year-old Chinese boy with kidney stones. We also recorded the patient’s clinical characteristics and treatments to enhance our understanding of the link between the *SLC3A1* genotype and the manifestations of cystinuria in the Chinese population.

## Materials and methods

### Study subjects

**Fig. 1:**
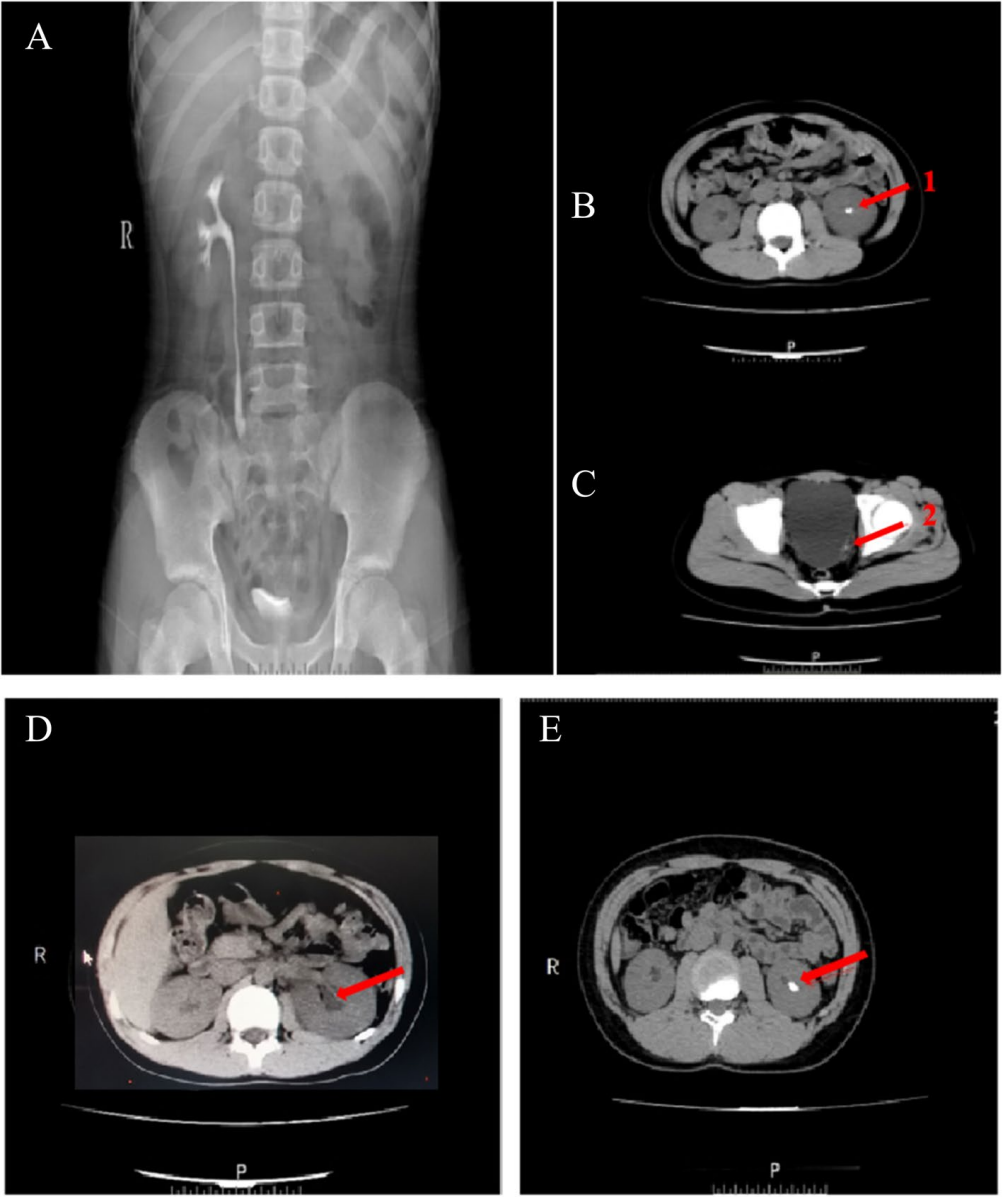
Radiographic findings in the patient. R: The right side of the body, P: The front of the body. **A** KUB film. **B** The red arrow indicates the stones in the left kidney (arrow 1); the average CT value of the arrow is 199.63 HU. **C** The red arrow indicates the stones in the left ureter (arrow 2). **D** Hydronephrosis was seen in the left kidney. **E** Six months later, residual fragments were found in his left kidney. The average CT value of the arrow is 752.25 HU. (The reference CT value for cystine stones is between 200 ~ 1100HU)

A 12-year-old Chinese boy was admitted for paroxysmal pain in the left lumbar region with no apparent cause. He was born in Pingdingshan, Henan, China, to a non-consanguineous family with no family history of genetic disease. He underwent a physical examination, liver and kidney function tests, and plain abdominal X-ray imaging of the kidney, ureter, and bladder (KUB). A physical examination showed a boy with normal development and neurological behaviors; his weight was 55 kg, height 155 cm, body temperature 36.4℃, and blood pressure 99/54 mmHg. A plain KUB x-ray showed stones and hydronephrosis in his left kidney (Fig. 1); most stones were relatively small, with two bigger ones measuring 0.3 × 0.2 cm and 1 × 0.9 cm (Fig. 1). The patient underwent extracorporeal shock wave lithotripsy and the smaller stones were passed successfully. Infrared spectroscopy indicated that the stones were made of cystine (Supplementary Fig. s1). We prescribed potassium citrate and tried to maintain the patient’s urinary pH > 7.5, but failed. Six months later, residual fragments were found in his left kidney and the stone had grown (Fig. 1E). Therefore, retrograde intrarenal surgery was used to remove the stones. In addition to potassium citrate, the patient was given tiopronin and sodium bicarbonate, which maintained a urinary pH > 7.5. The patient recovered and no additional kidney stones were detected. In this family, the proband was the only one with cystinuria-induced nephrolithiasis. His parents and brother were healthy, without any kidney abnormalities. After the patients’ parents signed the informed consent form, peripheral blood was drawn from the proband and his parents for genetic testing. His brother refused to be involved in the study.

### Whole-exome Sequencing (WES)

Genomic DNA was extracted from peripheral blood with a GenMagBio Genomic DNA Purification Kit (Changzhou, China) and fragmented using a Bioruptor® Pico (Diagenode, Ougrée, Belgium). We checked its quality with an Agilent 2100 Bioanalyzer and 4200 TapeStation System (Agilent Technologies, Inc., Santa Clara, CA, USA) and recorded the total DNA concentration with a NanoDrop™ One Spectrophotometer (Thermo Fisher Scientific, Waltham, MA, USA). A VAHTS™ Universal DNA Library Prep Kit for Illumina V3 (Vazyme Biotech, Nanjing, China) was used to build a DNA library. Capture and amplification were conducted with SureSelect Human All Exon V7 (Agilent Technologies, Inc.). Finally, the HiSeq 4000 system (Illumina, Inc., San Diego, CA, USA) was used to sequence the whole exome.

### Bioinformatics analysis

Trimmomatic-0.39 [2] was used to remove the sequencing adaptors and low-quality reads. The Burrow Wheeler Aligner (ver. 0.7.17-r1188) was used to align the cleaned reads to the human reference genome GRCh37 [16]. Genome Analysis Toolkit ver. 4 (GATK4) HaplotypeCaller was used to call single-nucleotide variants and small indels [6]. The variants were annotated by Vcfanno [20] based on the 1000 Genomes Project [11], Exome Aggregation Consortium (ExAC) [15], and gnomAD (v2.1) [17] annotation databases. We prioritized variants that occurred in known cystinuria-related genes *SLC3A1* and *SLC7A9* depending on the clinical diagnosis and phenotype information.

### Sanger sequencing

Sanger sequencing of *SLC3A1* (NM_000341.4) was performed on participating family members to determine whether the candidate variant co-segregated with the phenotype in the family. Two pairs of primers for c.898_905del and c.1898_1899insAT (Supplementary Table s3) were designed using NCBI Primer-BLAST and synthesized by ShangYa Bio Technology (Shanghai, China). The PCR products were purified using PCR purification kits (LifeSciences, Hangzhou, China) and sequenced on a SeqStudio Genetic Analyzer System sequencer (Applied Biosystems, Waltham, MA, USA).

## Results

### Laboratory findings

The patient’s initial renal and liver functions were normal, except for an elevated blood urea nitrogen (BUN) of 7.81 (normal 3.9–7.1) mmol/L and uric acid (UA) concentration of 512 (normal 150–440) μmol/L. Due to nephrolithiasis, the urine red blood cell count was 201 per μL and a bacterial infection was detected (Supplementary Table s1). After the lithotripsy and second operation, his BUN normalized and the UA concentration decreased gradually from 471 to 413 μmol/L (Table 1).

**Table 1:** Biochemical features of the patient before and after two treatments

Blood tests	BUN (mmol/L)	Cre (µmol/L)	UA (µmol/L)
First time	7.81	80	512
Second time	4.95	47	471
Third time	4.22	49	413
**Normal range**	**3.9–7.1**	**44–115**	**90–350**

### WES revealed two pathogenic variants of the SLC3A1 gene

WES generated 14.66G of original data with a Phred quality score of 30 (Q30) of 93.5%, and a mapping rate of reads to the human reference genome > 99.9%. The average sequencing depth was 100 × , with > 98% of the target sequence reaching 20 × .

We identified a novel compound heterozygous variant (c.[898_905del];[1898_1899insAT]) in the *SLC3A1* gene of this Chinese patient (Supplementary Table s2). Both mutations were frameshift variants: one was a deletion variant and the other an insertion variant. The deletion variant (NM_000341.4, c.898_905del, p.Arg301AlafsTer6) came from the mother and results in the formation of alanine instead of arginine, generating a stop codon after six amino acids. According to the standards and guidelines for interpreting sequence variants by the American College of Medical Genetics and Genomics (ACMG) [24], the variant is a frameshift variant leading to a null function. The null variant of the *SLC3A1* gene with an established loss of function is a known disease mechanism (PVS1 evidence); this variant has not been detected in the healthy population in the 1000 Genomes Project, ExAC, and gnomAD databases (PM2_supporting evidence). The patient’s phenotype was highly specific for the disease caused by *SLC3A1* (PP4 evidence). Therefore, we classified this variant as pathogenic.

**Fig. 2:**
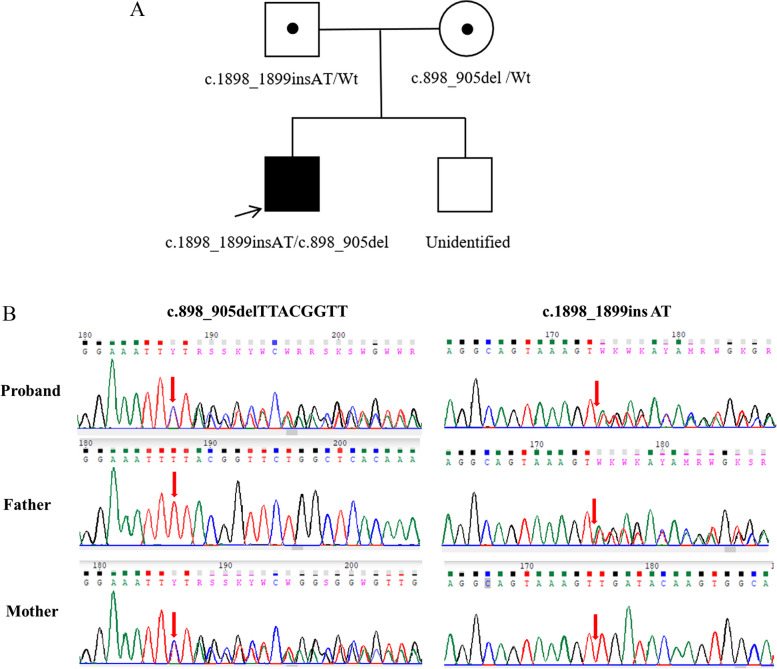
Pedigree and pathogenic variants in a Chinese family with cystinuria. **A** The family pedigree. The proband was the oldest boy in this family; his younger brother was not involved in the study. **B** Sanger sequencing revealed the *SLC3A1* gene mutations c.898_905del, and c.1898_1899insAT

The other mutation (NM_000341.4, c.1898_1899insAT, p.Asp634LeufsTer46) was inherited from the father and is a two-nucleotide insertion (AT) that generates leucine instead of aspartic acid and a stop codon after 46 amino acids. This variant is also a frameshift mutation leading to a loss of function (PVS1 evidence). The genotype frequency in the 1000 Genomes Project, ExAC, and gnomAD databases was 0.000 (PM2_supporting evidence). The patient’s phenotype is highly specific for the disease caused by *SLC3A1* (PP4 evidence). According to the ACMG guidelines, this variant (c.1898_1899insAT) was classified as pathogenic. Both variants were validated by Sanger sequencing of the proband and his parents (Fig. 2).

### Genotype and phenotype association

Some studies have found no obvious pattern between phenotype and genotype in cystinuria, which involves at least two genes [9, 18, 23]. Cystinuria caused by mutations in *SLC3A1* is classified as type A cystinuria, while mutations in *SLC7A9* cause type B. Most patients with cystinuria carry two mutations (AA, BB, or AB) [8]. Mutational analyses revealed biallelic *SLC3A1* mutations (AA genotype) in our proband. We observed that an older age of onset age in patients with the AA genotype. The proband in this study was first diagnosed with cystine stones at the age of 12 years, which is significantly older than the mean reported age of 5.59 years for cystinuria urolithiasis onset [1, 29]. The two variants carried by the proband were truncating mutations, which usually lead to a more severe phenotype. In our study, the BUN (mmol/L), creatinine (Cre; μmol/L), and UA (μmol/L) concentrations of the proband were all higher than the average values found in other study (Table 2) [27].

**Table 2:** Clinical comparison between our patient (WCZ) and 13 Chinese cystinuria patients (13C)

Sample	Age (years)	Stone locations	BUN (mmol/L)	Cre (μmol/L)	UA (μmol/L)
WCZ	12	Left kidney	7.81	80	512
13C	12.3 ± 4.9	76.9% in kidneys	4.5 ± 1.2	66.0 ± 26.9	348.6 ± 89.6

Age, age at which the first stone was identified

## Discussion

Cystinuria is a genetic disorder with high population heterogeneity globally. In a study of 13 Chinese cystinuria patients, Shen et al. [27] reported an average age at the first confirmed stone of 6.8 (range 0.8–16) years. Our study updates the clinical and genetic spectrum of cystinuria, and we have comprehensively studied the genetic mutations in *SLC3A1* that occurred in a Chinese family in which only a 12-year-old boy with thpe A cystinuria. We identified two heterozygous mutations (c.898_905del and c.1898_1899insAT) in *SLC3A1*; one of which has not been described in other cystinuria patients.

As of 2010, 133 mutations have been identified in *SLC3A1* [4] and the number is continuing to increase, but most identified to date are missense mutations [13, 27]. The deletion we found was in exon 5 and the AT insertion was in exon 10. Both variants alter the amino acids, leading to premature termination of protein translation(c.898_905del generating a stop codon after 6 amino acids and c.1898_1899insAT generating a stop codon after 46 amino acids) and disruption of gene function, and could be important in the rBAT coding protein functions, which is thought to be crucial in dibasic amino acid transepithelial transporters. The insertion variant c.1898_1899insAT identified in our case was reported in a Japanese patient in 2000 [9], the same study described four other mutations: three missense mutations (L346P, I445T, and C673R) and one deletion (1820delT). In a cohort of Japanese cystinuria patients, mutations in *SLC3A1* were found in around 10%, most of which were missense mutations [29]. In a study of 13 pediatric patients with cystine stones, Shen et al. [27] found 17 missense mutations in *SLC3A1* and *SLC7A9*; more than 90% (17/18) of these were missense mutations. It is uncommon to find two frameshift mutations in *SLC3A1* in Chinese cystinuria patients, and the deletion and insertion mutations in our study both caused frameshifts, but not a stop codon in the open reading frame. Our findings suggest that the genetic pattern of cystinuria is heterogeneous not only among different groups of people, but even within a local population.

Several researchers have examined the correlation between the clinical and genetic characteristics of cystinuria [13, 23,  27]. In our cystinuria patient, the BUN (mmol/L), creatinine (Cre; μmol/L), and UA (μmol/L) levels were all higher than the average values found in other studies (Table 2), we suspect that this may be related to the variant type of *SLC3A1* gene in the patient, as frameshift variants lead to more severe phenotypes than missense ones; after traditional treatments, including extracorporeal shock wave lithotripsy, alkalize urine and Oral medication treatment, these serum values normalized. Besides kidney stones and hydronephrosis, no other metabolic disorders were found in our patient, while in a study of 13 Chinese cystinuria patients, Shen et al. [27] found hyperoxaluria in 61.5%, hypercalciuria in 46.2%, hypocitraturia in 30.8%, and hyperuricosuria in 7.7%. Due to the limited number of cystinuria cases reported and analyzed in China, the correlation between *SLC3A1* mutation pattern and clinical presentation remains unclear. Rhodes et al. [23] found no association between the genotype and clinical course in cystinuria patients in the United Kingdom. Since cystinuria is caused by mutations in multiple genes, this makes it difficult to define the genetic pattern and etiology of cystinuria. Besides *SLC3A1* and *SLC7A9*, other genes can lead to similar presentations with cystine stones, such as *SLC7A13* and *PBX1* [18, 22]. Due to the complex genetic pattern of cystinuria, it is difficult to define a universal correlation between the phenotype and genotype. This study updates the clinical and mutational spectrum of cystinuria, especially in Chinese cystinuria patients.Therefore, broadening the spectrum of clinical and genetic variants could be crucial for the diagnosis and therapy of cystinuria in the long-term.

### Supplementary Information

**Additional file 1: Figure s1.** Infrared spectroscopy of the sediments revealed the crystal as cystine (observed spectrum of the stone material matched the cystine spectrum in a reference library) . An automatic infrared spectrum analysis system, LIIR-20 (approved by the Chinese FDA), was used in this study. T%, absorption frequency; WN, wavenumber.** Table s1.** Biochemical features of the patient with cystinuria (First time).** Table s2.** The pathogenicity classification of the *SLC3A1* variants.** Table s3.** The primers of *SLC3A1* variants for Sanger sequencing.

## Data Availability

The raw data are available from NODE (http://www.biosino.org/node) under accession number OEP000656. They are also available from the corresponding author upon reasonable request.

## References

[1] Alpay H, Ozen A, Gokce I, Biyikli N (2009). Clinical and metabolic features of urolithiasis and microlithiasis in children. Pediatr Nephrol.

[2] Bolger AM, Lohse M, Usadel B (2014). Trimmomatic: a flexible trimmer for Illumina sequence data. Bioinformatics.

[3] Calonge MJ, Chillaron GP (1994). Cystinuria caused by mutations in rBAT, a gene involved in the transport of cystine. Nat Genet.

[4] Chillaron J, Font-Llitjos M, Fort J, Zorzano A, Goldfarb DS, Nunes V, Palacin M (2010). Pathophysiology and treatment of cystinuria. Nat Rev Nephrol.

[5] D’Ambrosio V, Capolongo G, Goldfarb D. Cystinuria: an update on pathophysiology, genetics, and clinical management. Pediatr Nephrol. 2022;37(8):1705–11. 10.1007/s00467-021-05342-y. 10.1007/s00467-021-05342-y 34812923

[6] DePristo MA, Banks E, Poplin R, Garimella KV, Maguire JR, Hartl C, Daly M (2011). J. A framework for variation discovery and genotyping using next-generation DNA sequencing data. Nat Genet.

[7] Edvardsson VO, Goldfarb DS, Lieske JC, Beara-Lasic L, Anglani F, Milliner DS, Palsson R (2013). Hereditary causes of kidney stones and chronic Kidney Disease. Pediatr Nephrol.

[8] Eggermann T, Venghaus A, Zerres K (2012). Cystinuria: an inborn cause of urolithiasis. Orphanet J Rare Dis.

[9] Egoshi KI, Akakura K, Kodama T, Ito H (2000). Identification of five novel SLC3A1 (rBAT) gene mutations in Japanese cystinuria. Kidney Int.

[10] Font-Llitjos M, Jimenez-Vidal M, Bisceglia L, Di Perna M, de Sanctis L, Rousaud F, Nunes V (2005). New insights into cystinuria: 40 new mutations, genotype-phenotype correlation, and digenic inheritance causing partial phenotype. J Med Genet.

[11] Genomes Project C, Auton A, Brooks LD, Durbin RM, Garrison EP, Kang HM, Abecasis G (2015). R. A global reference for human genetic variation. Nature.

[12] Goldfarb DS (2011). Potential pharmacologic treatments for cystinuria and for calcium stones associated with hyperuricosuria. Clin J Am Soc Nephrol.

[13] Kim JH, Park E, Hyun HS, Lee BH, Kim GH, Lee JH, Cheong H (2017). Genotype and Phenotype Analysis in Pediatric patients with Cystinuria. J Korean Med Sci.

[14] Kummer S, Venghaus A, Schlune A, Leube B, Eggermann T, Spiekerkoetter U (2014). Synergistic mutations in SLC3A1 and SLC7A9 leading to heterogeneous cystinuria phenotypes: pitfalls in the diagnostic workup. Pediatr Nephrol.

[15] Lek M, Karczewski KJ, Minikel EV, Samocha KE, Banks E, Fennell T, Exome Aggregation C (2016). Analysis of protein-coding genetic variation in 60,706 humans. Nature.

[16] Li H (2013). Aligning sequence reads, clone sequences and assembly contigs with BWA-MEM. Oxf Univ Press.

[17] MacArthur DG, Balasubramanian S, Frankish A, Huang N, Morris J, Walter K, Tyler-Smith C (2012). A systematic survey of loss-of-function variants in human protein-coding genes. Science.

[18] Olschok K, Vester U, Lahme S, Kurth I, Eggermann T (2018). No evidence for point mutations in the novel renal cystine transporter AGT1/SLC7A13 contributing to the etiology of cystinuria. BMC Nephrol.

[19] Palacín M, Nunes V, Font-Llitjós M, Jiménez-Vidal M, Fort J, Gasol E, Pineda M, Feliubadaló L, Chillarón J, Zorzano A (2005). The genetics of heteromeric amino acid transporters [J]. Physiology (Bethesda, Md).

[20] Pedersen BS, Layer RM, Quinlan AR (2016). Vcfanno: fast, flexible annotation of genetic variants. Genome Biol.

[21] Popovska-Jankovic K, Tasic V, Bogdanovic R, Miljkovic P, Baskin E, Efremov G, Plaseska-Karanfilska D (2009). Five novel mutations in Cystinuria genes SLC3A1 and SLC7A9. Balkan J Med Genet..

[22] Reis ST, Leite KRM, Marchini GS, Guimaraes RM, Viana NI, Pimenta RCA, Mazzucchi E (2019). Polymorphism in the PBX1 gene is related to cystinuria in Brazilian families. J Cell Mol Med.

[23] Rhodes HL, Yarram-Smith L, Rice SJ, Tabaksert A, Edwards N, Hartley A, Coward RJ (2015). Clinical and genetic analysis of patients with cystinuria in the United Kingdom. Clin J Am Soc Nephrol.

[24] Richards S, Aziz N, Bale S, Bick D, Das S, Gastier-Foster J, Committee AL (2015). Q. A. standards and guidelines for the interpretation of sequence variants: a joint consensus recommendation of the American College of Medical Genetics and Genomics and the Association for Molecular Pathology. Genet Med.

[25] Saeedeh F, Saiedeh A, Kheirolahi M, Mohammadi M (2017). A novel mutation in SLC7A9 gene in Cystinuria. Iran J Kidney Dis.

[26] Sahota A, Tischfield JA, Goldfarb DS, Ward MD, Hu L (2019). Cystinuria: genetic aspects, mouse models, and a new approach to therapy. Urolithiasis.

[27] Shen L, Cong X, Zhang X, Wang N, Zhou P, Xu Y, Gu X (2017). Clinical and genetic characterization of Chinese pediatric cystine stone patients. J Pediatr Urol.

[28] Strologo LD, Pras E, Pontesilli C, Beccia E, Ricci-Barbini V, de Sanctis L, Rizzoni G (2002). Comparison between SLC3A1 and SLC7A9 cystinuria patients and carriers: a need for a new classification. J Am Soc Nephrol.

[29] Watanabe Y, Abe Y, Sakamoto S, Morimoto E, Taki Y, Hibino S, Watanabe S (2019). Pediatric Cystinuria Patient with Novel Mutation in SLC3A1. Glob Pediatr Health.

[30] Weinberger A, Rabinovtz SO, Brosh M, Adam S, De Vries A (1974). High frequency of cystinuria among Jews of Libyan origin. Hum Hered.

[31] Zhang Z, Zheng R, Zhu C, Geng H, Xu G (2022). Lipidomics characterization of the lipid metabolism profiles in a cystinuria rat model: Precalculus damage in the kidney of cystinuria. Prostaglandins Other Lipid Mediators..

[32] Yuen YP, Lam CW, Lai CK, Tong SF, Li PS, Tam S, et al. Heterogeneous mutations in the SLC3A1 and SLC7A9 genes in Chinese patients with cystinuria. Kidney Int. 2006;69(1):123–8. 10.1038/sj.ki.5000003. 10.1038/sj.ki.5000003 16374432

