# Supplementary material for: Novel compound heterozygous pathogenic variants in the SLC3A1 gene in a Chinese family with cystinuria
Source: BMC Med Genomics. 2023 Dec 19;16:333. doi: 10.1186/s12920-023-01767-6 (PMC10731833; doi:10.1186/s12920-023-01767-6)
Supplement: Supplementary file 1 — Additional file 1: Figure s1. Infrared spectroscopy of the sediments revealed the crystal as cystine (observed spectrum of the stone material matched the cystine spectrum in a reference library) . An automatic infrared spectrum analysis system, LIIR-20 (approved by the Chinese FDA), was used in this study. T%, absorption frequency; WN, wavenumber. Table s1. Biochemical features of the patient with cystinuria (First time). Table s2. The pathogenicity classification of the SLC3A1 variants. Table s3. The primers of SLC3A1 variants for Sanger sequencing. [file 12920_2023_1767_MOESM1_ESM.docx]

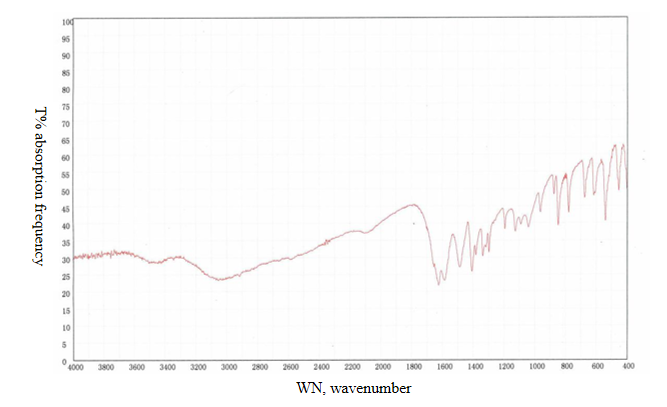


Figure s1. Infrared spectroscopy of the sediments revealed the crystal as cystine (observed spectrum of the stone material matched the cystine spectrum in a reference library) . An automatic infrared spectrum analysis system, LIIR-20 (approved by the Chinese FDA), was used in this study. T%, absorption frequency; WN, wavenumber.

Table s1. Biochemical features of the patient with cystinuria (First time)

| Blood tests |  |  | Normal range |
| --- | --- | --- | --- |
|  | WBC (× 10^9^/L) | 6.11 | 5-12 |
|  | PLT (× 10^9^/L) | 282 | 100-400 |
|  | NEUT(× 10^9^/L) | 4.24 | 1.8-6.3 |
|  | LYMPH(× 10^9^/L) | 1.40 | 1.1-3.2 |
|  | MONO (× 10^9^/L) | 0.42 | 0.1-0.6 |
|  | EO (× 10^9^/L) | 0.02 | 0.02-0.52 |
| Renal function  and Liver Function | BUN (mmol/L) | 7.81 | 3.9–7.1 |
|  | eGFR (ml/min) | 155.39 | 90~120 |
|  | Cr (µmol/L) | 80 | 44–115 |
|  | UA (µmol/L) | 512 | 90–350 |
|  | TBIL (µmol/L) | 12.5 | 0-23 |
|  | DBIL (µmol/L) | 5.1 | 0-6.8 |
|  | TP (g/L) | 76.4 | 65-85 |
|  | GLO (g/L) | 32.7 | 20-40 |
|  | ALT (U/L) | 12 | 9-50 |
|  | AST (U/L) | 17 | 15-40 |
|  | AST/ALT | 1.4 | 0.9-1.2 |
| Electrolyte analysis | K^+^ (mmol/L) | 4.63 | 3.5-5.3 |
|  | Na^+^ (mmol/L) | 137 | 137-147 |
|  | Cl^−^ (mmol/L) | 102.5 | 99-110 |
|  | Ca^2+^ (mmol/L) | 2.43 | 2.11-2.52 |
|  | Mg^2+^ (mmol/L) | 0.97 | 0.75-1.02 |
|  | P (mmol/L) | 2.06 | 0.85-1.51 |

Table s2. The pathogenicity classification of the *SLC3A1* variants.

| Transcript | c.Change^a^;p.Change^b^ | Zygosity  (Segregation) | gnomAD^c^ | Evidence of pathogenicity | ACMG^d^ classification |
| --- | --- | --- | --- | --- | --- |
| NM_000341.3 | c.898_905del;  p.Arg301AlafsTer6 | het(p,wt;m,het) | None | PVS1,PM2_supprorting,PP4 | Pathogenic |
| NM_000341.3 | c.1898_1899insAT;  p.Asp634LeufsTer46 | het(p,het;m,wt) | None | PVS1,PM2_supporting,PP4 | Pathogenic |

Note. c.Change, nucleotide change; del, deletion; ins, insertion; fs, frameshift; p.Change, amino acid change; het, heterozygous; p, paternal; m, maternal; wt, wildtype; PVS, Very strong evidence of pathogenicity; PM, Moderate evidence of pathogenicity; PP, Supporting evidence of pathogenicity.

^a^ Impact of variant on cDNA level.

^b^ Impact of variant on the amino acid or protein level.

^c^gnomAD, variant frequencies listed for homozygous/hemizygous (if applicable)/heterozygous/total alleles (http://gnomad.broadinstitute.org/).

^d^ACMG, American College of Medical Genetics and Genomics Standards and Guidelines Classification as pathogenic, likely pathogenic, or VUS (Richards Genet Med 17(5):405, 2015)

Table s3. The primers of *SLC3A1* variants for Sanger sequencing.

| *SLC3A1* Mutation | Forward primer | Reverse primer |
| --- | --- | --- |
| c.898_905del | 5’-TGTGGGAGTCGCTAAATGCAG -3’ | 5’- TGGATCTCATCTCTCAGGTGC -3’ |
| c.1898_1899insAT | 5’- GGGCTGGTTTTGCCATTTGA -3’ | 5’- AAGCTGTTTGGCGATGAAGG -3’ |
